# Physical activity in patients with venous leg ulcer – between engagement and avoidance. A patient perspective

**DOI:** 10.1177/0269215510371424

**Published:** 2011-03

**Authors:** Kirsti Skavberg Roaldsen, Gabriele Biguet, Britt Elfving

**Affiliations:** Karolinska Institutet, Department of Neurobiology, Care Sciences and Society, Division of Physiotherapy, Stockholm, Sweden and Sunnaas Rehabilitation Hospital, Oslo, Norway; Karolinska Institutet, Department of Neurobiology, Care Sciences and Society, Division of Physiotherapy, Stockholm, Sweden

## Abstract

**Objective**: To identify and describe the qualitative variations in how physical activity is perceived and understood by individuals with current or previous venous leg ulcer.

**Design**: A qualitative study using semi-structured interviews.

**Method**: Twenty-two individuals aged 60–85 years were interviewed. The interviews were recorded, transcribed verbatim and analysed by three researchers using a phenomenographic research approach. A set of categories of descriptions and their internal relationships were constructed based on the essential features of the variation in patients’ perceptions of physical activity.

**Results**: Four categories of descriptions were identified: (i) ‘self-management’, (ii) ‘instructions and support’, (iii) ‘fear of injury’ and (iv) ‘a wish to stay normal’. The categories could be interpreted by a two-dimensional construct: (1) perception of venous leg ulcer as a chronic or acute condition and (2) engagement or avoidance behaviour toward physical activity. Chronicity and behaviour combined together formed a 2 × 2 square housing the four qualitatively different categories. Irrespective of category, the participants reported that information given by caregivers regarding leg ulcer and physical activity was insufficient or contradictory. Written information or exercise programmes were not obtained regularly and not at all in primary care.

**Conclusion**: A dichotomous view emerged from participants’ experiences of physical activity based on (1) perception of venous leg ulcer as a chronic or acute condition and (2) engagement or avoidance behaviour toward physical activity.

## Introduction

Patients with venous leg ulcers have been reported to be less physically active than age-matched controls,^1,2^ which is worrying as these patients seem to have an increased risk of disease-related comorbidity.^3^ Pain and functional limitations seem to persist despite wound healing.^1,4^ Therefore, physical activity in combination with compression therapy is recommended, reversing the effects of venous hypertension, decreasing wound-healing time, and preventing wound recurrence and post-ulcer functional limitations.^5–7^ Leg exercise and walking have been found to effectively stimulate the calf-muscle pump which supports venous circulation and enhances venous return (pumping of blood from the leg towards the heart).^8–10^ Thus, patients with venous disease might gain additional benefits from physical activity.

According to a review by Persoon *et al.*,^3^ low levels of physical activity in patients with chronic venous disease are caused primarily by pain, but also result from bulky wound dressings, swollen legs, leaking wounds, high age and the need to wear large shoes.^3^ In a previous study we found that fear of movement and associated avoidance behaviour should also be considered, as fear-avoidance beliefs were a better predictor of low physical activity in these patients than pain.^11^ Fear of exposing the leg to strain, and avoidance of situations that aggravate pain, such as standing, walking and going out, may hypothetically affect physical activity negatively. Such behaviour has been reported in patients with venous ulceration.^12,13^

Physical activity can be a demanding and often complex behaviour with known poor adherence.^14^ To accomplish changes in lifestyle and physical activity it is important to explore the different ways patients perceive and comprehend physical activity, as this influences the way they act. Although physical activity is recommended for patients with leg ulcer, to characterize variations in how physical activity is perceived and understood have not previously been studied. A qualitative research approach was chosen for this study for two reasons. First, the aim of the study was to explore the patient perspective, which favours a qualitative approach. Second, the qualitative approach is helpful for understanding the complexities of human health and behaviour.^15^ Thus, the aim of this study was to identify and elucidate the qualitative variations in how physical activity is perceived and comprehended in individuals with current or previous venous ulcer.

## Methods

To ascertain the qualitative variations in individuals’ perceptions of physical activity, semi-structured interviews were conducted and analysed using a phenomenographic research approach.^16,17^ Phenomenography assumes that there is a limited number of qualitatively different ways of understanding or experiencing phenomena which are shared by different people in a similar situation. The outcome of a phenomenographic research approach is a set of logically related categories that outline the variation in peoples’ perceptions of the phenomenon. These categories and the logical relations between them provide the final outcome space for the research.^18–20^

### Subjects and procedure

All patients aged 60–85 years with chronic venous insufficiency and current or previous leg ulcers, from the Department of Dermatology, Karolinska University Hospital, Stockholm, Sweden, who had participated in a previous study (*n =* 98) and had given permission to be contacted again (*n =* 69), were informed by letter and asked to participate. Forty-seven answered the letter (68%); 29 accepted the invitation (42%) while 18 declined or wanted additional information. In addition, two patients were recruited directly from the Department of Dermatology. All eligible patients were telephoned by the first author and given additional information about the study and the method. During these telephone conversations two patients were excluded because of cognitive impairments, leaving 29 in the eligible group. The first author scheduled interview meetings 2–10 days ahead of the interviews. The recruitment ended when no new aspects of the phenomenon were stated in the interviews. Thus, finally the study sample comprised 22 out of the 29 eligible patients (Table 1).

**Table 1:** Sociodemographic-, disease- and health-related data for 22 individuals with venous leg ulcer

Gender: female/male, *n* (%)	13/9 (59/41)
Age: years, median and range	75 (60–85)
Married/cohabitant: *n* (%)	12 (55)
Occupational status: *n* (%)	
Full-time work	2 (9)
Sickness pension	1 (5)
Old age pension	19 (86)
Disease duration: years, median and range	8 (0.08–51 years)
Number of chronic conditions: median and range	4 (2–7)
Ulcer status: healed/active, *n* (%)	12/10 (55/45)
Compression therapy: yes/no, *n* (%)	17/5 (77/23)
Walking aids: yes/no, *n* (%)	9/13 (41/59)
Community services: yes/no, *n* (%)	6/16 (27/73)
Pain in last week: *n* (%)^a^	
None or mild	8 (36)
Discomforting or distressing	14 (64)
Horrible or excruciating	0
Is pain a problem? yes/no, *n* (%)	8/24 (36/64)
Self-rated mobility: *n* (%)	
Poor	0
Fair	10 (45)
Good	11 (50)
Excellent	1 (5)
Level of physical activity: *n* (%)^b^	
Hardly any physical activity	2 (9)
Mostly sitting, sometimes a walk; sometimes light domestic work	2 (9)
Light physical exercise 2–4 hours/week; responsibility for light domestic work	12 (55)
Moderate exercise 1–2 hours/week; responsibility for all domestic work	6 (27)
Moderate exercise for 3 hours/week	0
Hard or very hard exercise regularly and several times/week	0
Body Mass Index: *n* (%)	
Normal (18.5–24.9)	14 (64)
Overweight (25–29.9)	5 (23)
Obese (30–34.9)	3 (14)

a The six-point verbal rating scale for pain assessment.^21^

b A six-point scale for assessing physical activity, including household activities.^22^

An interview guide in line with the phenomenographic approach was developed and tested by the authors. The questions focused on experiences and understanding of physical activity in relation to leg ulcer, where and how the patients had got information on physical activity and what advice they themselves would give to other patients with venous leg ulcers and caregivers regarding physical activity (Table 2). Physical activity was defined as all daily activities that increased metabolism, such as household activities, shopping, transport, walking, exercising, etc. Two pilot interviews were performed to train interview technique and to test and modify the guide. The pilot interviews were included in the analysed data. All the interviews were conducted by the first author within five weeks during May and June 2008. Two were done at the Karolinska University Hospital, the others in the patients’ homes. The interviews were recorded on a digital voice recorder (Olympus DS-2300) and lasted between 26 minutes and 1 hour 23 minutes.

**Table 2:** The interview guide (translated from Swedish)

Would you like to tell me briefly what having leg ulcer means to you?
What does the concept physical activity mean to you?
In what ways are you physically active these days?
What’s it like for you to be physically active with your leg ulcer and in view of your general health state?
Tell me how physical activity affects you – both positive and negative experiences of physical activity in view of your leg ulcer.
What motivates you to be physically active?
What stops you from being physically active?
What information have you received about physical activity?
Is there any difference how physically active you are now compared to before you got leg ulcer trouble?
What advice would you give to other patients with similar trouble about physical activity and leg ulcer?
What advice would you give to doctors and nursing staff about physical activity and leg ulcer?
What information have you got about physiotherapy?
What are your thoughts about the future?
Have you got any new thoughts after our discussion of physical activity that you want to add?
Do you think we’ve discussed things that you feel are important for you about physical activity?

Written and oral information was given that participation was voluntary, that confidentiality was guaranteed and that the participants were free to withdraw at any time without consequences. The participants gave their informed consent prior to the interviews. Following the interviews, participants were given treatment and exercise advice based on the information given in the interviews. Ethical approval was obtained from the Regional Ethical Review Board in Stockholm (D no. 04-565/2).

### Data analysis

All 22 interviews were recorded and transcribed verbatim (Olympus AS-2300 PC transcription kit) and printed out, 15 by the first author and 7 by a secretary. Based on the interviewer’s logbook and by listening to the interview files several times, the 15 most information-rich interviews were selected for analysis by all three authors. The remaining seven were analysed only by the first author. These seven gave no new information, but contributed well to the existing result and all 22 interviews were included in the analysis. The transcripts, consisting of 330 pages, were analysed following the seven steps of the phenomenographic analysis procedure described by Dahlgren and Fallsberg.^16^ To ensure validity in the analysis process, all three authors conducted the analysis of two transcripts, participant (P) P3 and P19, first separately and then together. Consensus was reached before the remaining transcripts were analysed. Finally, an uninitiated researcher checked the results for clarity.

1. *Familiarization.* Each transcript was read several times in order to become familiar with and to gain a general picture of the material.
2. *Condensation.* A short summary of each interview was established using questions which were useful to identify significant aspects of the studied phenomenon. Responses from all the patients to these certain questions were compiled. Thereafter the most significant elements in the descriptions from each patient were identified together with the statements that corresponded to the aim of the study. In this way the material was reduced to 59 significant preliminary themes.
3. *Comparison.* The preliminary themes and their associated statements were compared to find similarities and differences in content.
4. *Grouping. *In this step variations emerged. Seven preliminary categories of descriptions were identified by grouping themes with similar content: (1) physical activity (PA) as a part of life, as a part of the disease; (2) PA as a treatment strategy; (3) PA as dilemma, ambivalence and fear; (4) PA as a ‘must’, as a prescription to ease bad conscience; (5) PA as a strategy to divert attention from symptoms; (6) PA as a reminder of dysfunction; (7) PA demand knowledge, support, directions, participation, adaptations, etc. These categories were discussed several times by all authors to avoid overlap. They were cross-checked with the content of the original interviews before the analysis was considered satisfactory. The goal was to arrive at a point where, despite further cross-checking, each category remained stable. Finally the seven preliminary categories were synthesized into four discrete categories of descriptions. These categories are decontextualized and describe the essential features of the variation.
5. *Articulating.* Each category of description was described after negotiated agreement and illustrated by quotations selected from the interviews.
6. *Labelling.* The categories were ‘labelled’ with a suitable metaphor.
7. *Contrasting.* The categories were compared to find a structure that could relate them to each other.

## Results

**Figure 1:**
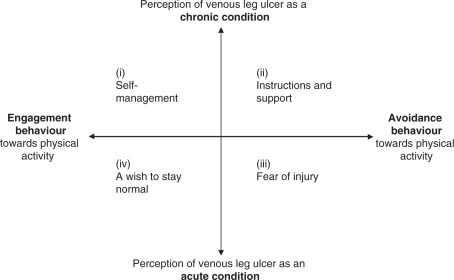
The internal relationship between the categories of descriptions of the phenomenon physical activity in patients with venous leg ulcer (*n =* 22).

A two-dimensional construct emerged from participants’ experiences of physical activity based on (1) perception of venous leg ulcer as a chronic or acute condition and, (2) engagement or avoidance behaviour toward physical activity. Chronicity and behaviour combined together formed a 2 × 2 square housing four qualitatively different categories of perception and comprehension of physical activity which emerged from the phenomenographic approach: (i) ‘self-management’, (ii) ‘instructions and support’, (iii) ‘fear of injury’ and (iv) ‘a wish to stay normal’ (Figure 1). These were categories of ideas illuminating four different ways that patients might think about physical activity, and not categories of patients.

Irrespective of category, physical activity was experienced as a positive factor in life, however limited either directly by barriers such as pain, swelling, chafing bandages, running sores, problems with shoes and fear of injury; or indirectly through multimorbidity and old age or lack of time due to time-consuming ulcer care and care of family members. Barriers to physical activity did not differ among the categories, although ways of confronting them changed. Further, the participants reported that information from caregivers regarding physical activity and leg ulcer was insufficient or contradictory. Written information or exercise programmes were not obtained regularly and not at all in primary care. ‘Live as usual’ was the most explicit advice given by health care providers. Thus most participants displayed poor understanding of the effect of physical activity on venous disease. Physiotherapy was a treatment option only for those who had been referred to hospital for skin grafts*; *it was not offered to patients treated in primary care.

### Physical activity as (i) ‘self-management’

In this category individuals understood the chronic nature of venous disease as a cause of leg ulcer, and physical activity was seen as a life-long treatment strategy to increase circulation. Hence, physical activity called for self-management, a personal initiative and individual responsibility. Physical activity was described as a necessity to be able to live with the disease and as an important factor to regain and maintain functioning and health despite the inconvenience of venous disease. Physical activity gave a feeling of taking responsibility and being involved in the treatment. Personal involvement and an explicit goal made physical activities meaningful, enjoyable and worthwhile despite barriers. Knowledge about physical activity and positive experience earlier in life seemed to motivate and become a driving force. For some, even fear became a driving force as inactivity meant the possibility of amputation and dependence on a wheelchair. Citations:

The only way is to keep moving and improve your circulation. And then you improve everything in your body. (P 9)

You can ask yourself, ‘d’you want to get better and get as close as possible to when you were well’, it costs you some effort, both physical and mental. And for that effort I’m only grateful to get help. (P 16)

I have to walk or else my legs will die on me…I saw what they were like when I’d been bedridden a few months. This thigh, the leg I didn’t use, shrank. You see how quickly it happens … that’s why I don’t want to sit in a wheelchair. (P 1)

When I got to the hospital the first thing that happened was ‘take this wheelchair, you can have it’. ‘No thanks,’ I said, ‘I don’t want a wheelchair.’ I was frightened it would become permanent. I didn’t want to be locked in that thing. (P 16)

The lack of explicit and clear-cut information regarding physical activity was experienced as confusing. However, these individuals chose not to be pacified, despite a feeling of uncertainty and disobedience when being physically active when told, for example, not to exercise with an open ulcer. As no long-term negative effects of physical activity were experienced, the individuals chose to trust themselves and stay physically active. When barriers to activity arose, they found ways to cope. Physical activity called for compression therapy, painkillers and, for some, assistive devices, which were used inventively. A walking frame on wheels was not only a walking device, but also a protective one, for avoiding possible leg injury by holding people and items at a distance. Bandages and compression stockings were used not only as compression devices, but also as a protective second skin. To enhance physical activity, step counters, Nordic walking sticks, group activities and help from colleagues, friends and family were used.

The doctors said, ‘No, no strenuous activity as long as you’ve got open ulcers … it would hamper the healing of the ulcer’ … Well, I suppose I question the doctors and stuff, but not if they can give me an adequate reason. But they can’t. (P 1)

I think I’d got three nurses there, one was indifferent, the second said ‘of course you can move about, go on running’, and the third said ‘walk at a slow pace’. I was confused. (P 1)

I’ve found that my leg kind of gives way, it couldn’t manage properly when things were worst. But then a painkiller tablet was a help… . Well, you get a breathing space, makes things easier actually. … Painkillers are some help to self-help, you could say. (P 17)

I’m very grateful for these aids, walking frames and so on. Without it I don’t think I’d be able to walk. It’s an unbelievably good aid. And it’s gentle, for it protects your legs when you’re walking. Perfect, I reckon. (P 3)

The compression stockings are like my second skin. They are a protection when I bump into something. (P 3)

### Physical activity as (ii) ‘instructions and support’

In this category the chronic nature of venous disease and the importance of physical activity to increase circulation were understood. However, individuals lacked the ability to initiate or perform exercise on their own. Physical activities required external support and reminders, as individuals lacked initiative or discipline or were overwhelmed by barriers. The lack of initiative and discipline was reinforced by insufficient and contradictory information. Physical activity as a treatment strategy was understood as the responsibility of the caregivers, leading to a wish for someone else to take the responsibility or at least share the responsibility with the individuals. They were disappointed in health care providers who focused solely on ulcer care and not on the patient’s well-being.

No, no. They didn’t say anything about what I could do myself. … There was just concentration on getting rid of that sore. (P 19)

The patient isn’t encouraged to contribute himself in any way… . If the patient doesn’t realize what he or she can do, even the best doctor’s no help. (P 22)

When you turn up for treatment only for your leg you could be pushed a bit. I think you’re at your most receptive then… . They look at sores and change bandages and do what they can with that and don’t bother so much about the rest of the person. (P 14)

Individuals in this category want to follow given advice, and clear written exercise programmes, adapted and adjusted for each one, preferably including instructions. Physical activity should be prescribed, not only generally recommended. Individuals demonstrated the belief that frequent encouragement and reminders would facilitate physical activity.

Not just changing the bandage but ‘do so-and-so, this-and-that exercise’. Then get a leaflet with exercises and illustrations. And then people will do this. They want to get rid of their ulcers. No one thinks they’re any fun. This should be a must. (P 22)

Actually I’m a bit lazy, I’d need someone to say ‘now it’s ten o’clock, now we’re going for a walk’. Someone who gets you going, you get … once the ulcer has healed, then it’s fine. (P 3)

And once again it was the physios that nagged [laughter]. We need that nagging, it’s good. (P 16)

### Physical activity as (iii) ‘fear of injury’

In this category physical activity involved fear of increased pain and fear of injury. Leg ulcer was experienced as an acute condition that would disappear once the ulcer had healed. When leg ulcers occurred the individuals had problems understanding why this was happening to them. Responsibility for treatment was handed over to the specialists. Pain was understood as a threat to healing, resulting in avoidance of activities, and the use of painkillers.

It seemed strange that I got skin changes… . So I’m a bit surprised why there’s suddenly come an ulcer too, but that must be because of my circulation as far as I know… . I’m hopin to get help to get rid of it as quickly as possible. (P 2)

I thought nothing could be done. You rely on them to do the right things. (P 17)

My leg is in the way. … So I go by car. Then I just don’t bother to walk at all. (P 17)

My doctor said it’d heal worse if it hurt, so I avoid moving about when it hurts. (P 2)

Individuals were preoccupied by fear that activity will increase pain and harm the leg, thus physical as well as social activities were viewed as risk-taking action to be postponed until the ulcer was healed. To prevent injury, the individuals used their skill in recognizing threats known to damage the skin on their legs. When interacting with other people, they were watchful and preoccupied with their own safety and protection.

I suppose I’ve always been a little scared. The fear is there, latent, underneath, at the back of my head, saying ‘be careful’… . Don’t go and fall down, take your stick, be careful, like. … Perhaps it’s important to keep the fear, so you don’t hurt yourself. (P 3)

Oh yes, I’m ever so careful. Definitely. If there’s too many people, I don’t join in. Well, if I get kicked or something happens, then it’s likely I’ll get an ulcer again. So I’m very, very careful. (P 17)

I get frightened and at the same time I’m cross with myself for being frightened, so I’ve not gone for walks. Then you sit there of an evening, now nothing happened today either. The ulcer was the part of my body that didn’t like walking. (P 22)

### Physical activity as (iv) ‘a wish to stay normal’

In this category each recurrent leg ulcer was perceived as a single, acute episode. Furthermore, having a leg ulcer threatened the identity as a healthy person and the individuals did not want anybody to know about their condition. They wanted to remain normal without medical aids or limitations. Physical activity was primarily motivated by the fact that activity helped the individuals to maintain an identity as a normal person.

Many of my neighbours here don’t know I’ve got leg ulcers. Why should I tell them? I don’t want anyone to feel sorry for me. Even the bloke I sometimes go fishing with doesn’t know. (P 5)

You don’t think so much, it just is. I just think it’ll sort itself out sometime, it has to heal eventually. It can’t go on like this for ever. (P 7)

You have to dare. You can’t be scared. You gain a lot by daring [laughter]. You shouldn’t let anything decide over you, even ulcers. (P 7)

Physical activity was used to gain well-being and distract pain, not as a treatment strategy.

Adherence to compression therapy was poor and inconsistent, given the lack of knowledge of the chronic nature of the underlying disease and the fact that using compression stockings threatened one’s identity as a normal person. Some of the individuals also feared that compression stockings and bandages limited motion and leg muscle activity.

My advice is to try and find some physical activity that is enjoyable. … Some people think gymnastics is fun, I think it’s frightful … and that going for walks is dead boring. But working in the garden, that’s what I think is fun. Being able to work in your garden is therapy through the grace of God … if it hurts a bit, it’s a good thing to get over it. Exercise, walk or something. (P 13)

Compression stockings is something I don’t want to know about. I wasn’t quite alert enough, it just wasn’t my style to have compression stockings. Elderly ladies were the ones who had them [laughter]. (P 16)

It’s just that you can’t have compression stockings on for too long, for the muscle becomes a bit limp, see … perhaps the stocking hinders the actual muscle a bit … when you don’t have anything on it, the foot can move a bit more easily, because of the muscle and tendon. It may be that the muscle gets too relaxed and that can’t be a good thing either. (P 9)

## Discussion

The present study addresses the perceived utility of physical activity in the context of venous leg ulcer in a group of 22 patients living with chronic venous insufficiency. A two-dimensional construct emerged from participants’ experiences of physical activity based on (1) perception of venous leg ulcer as a chronic or acute condition, and (2) engagement or avoidance behaviour toward physical activity. Chronicity and behaviour combined together forms a 2 × 2 square housing four qualitatively different categories of perception and comprehension of physical activity that patients with venous leg ulcer might hold: (i) ‘self-management’, (ii) ‘instructions and support’, (iii) ‘fear of injury’ and (iv) ‘a wish to stay normal’.

Barriers to physical activity such as pain, chafing bandages and problems with shoes were experienced and expressed by the participants irrespective of way of thinking about physical activity (category). Information regarding the importance and benefits of physical activity as a treatment strategy was not given as routine and physiotherapy was not presented as a treatment option in primary care. Hence, understanding of the potential effect of physical activity and exercise on ulcer-healing and the prevention of recurrence was generally poor, as was understanding of the importance of adherence to compression therapy to optimize the effect of physical activity on venous circulation. These findings are consistent with those of Edwards *et al.*,^23^ who reported that patients’ knowledge of their condition was poor, with little understanding of the underlying pathology.

Whether or not leg ulceration was viewed as a chronic or an acute event was an important aspect of various ways of perceiving and understanding physical activity. The participants who understood the chronic nature of venous insufficiency as the cause of leg ulcer saw physical activity as a plausible treatment strategy, as seen in the categories ‘self-management’ and ‘instruction and support’. This was in contrast with patients who conceived leg ulceration as an acute event, as in ‘fear of injury’ and ‘a wish to stay normal’. These findings are consistent with those of Flaherty,^4^ who found that participants who were fairly well-informed made conscious decisions about life-style changes, including exercise, whereas participants who displayed poor understanding of the disease and the treatment, especially of the chronicity of venous insufficiency, viewed their previous ulceration as an acute episode that had healed and considered themselves cured.

Briggs and Flemming^24^ suggested that a change in care focus was required, among both health care professionals and patients, from viewing leg ulceration as an acute event, to viewing it as a chronic condition with needs comparable to those of other chronic conditions. The shift from specialists’ healing rates to self-care and symptom management (reducing pain and improving mobility) may in turn affect healing, as also argued by Persoon *et al.*^3^

Irrespective of category, few of our participants had been encouraged by health care providers to consider physical activity as an optional treatment strategy or to find ways to exercise despite the inconveniences of venous insufficiency and recurrent leg ulcer. This is worrying, as clinical guidelines state the importance of encouraging mobility and physical activity to prevent ulcers.^25,26^ Vague and inaccurate lifestyle advice such as ‘live as usual’ and contradictory exercise advice, as found through the present study, are not fruitful strategies. This was especially evident in patients who felt that physical activity was the responsibility of health care providers – as in the category ‘instruction and support’ – and patients who felt that physical activity was risk-taking – as in ‘fear of injury’.

Health professionals’ ability to impart clear-cut and consistent information must be strongly improved, likewise their ability to support and encourage physical activity. However, as demonstrated by our participants in the categories ‘fear of injury’ and ‘a wish to stay normal’, some patients are reluctant or unable to absorb information. This in turn may make them deny that there is anything wrong, as in ‘a wish to stay normal’ or to wrongly believe and expect that physical activity will cause injury – ‘fear of injury’.

In our study, the perception of physical activity was closely related not only to the ‘chronic–acute’ dimension (Figure 1), but also to personal factors such as initiative, discipline, self-confidence, skills, past experience, health beliefs, motivation, insecurity and fear. We named this dimension ‘engagement–avoidance’ as these personal factors influence the ways barriers and limitations to physical activity are confronted or avoided.

Insecurity and lack of initiative and/or discipline, as in ‘instructions and support’, could be related to lack of self-motivation for exercise, which is negatively associated with physical activity.^27^ Without initiative and discipline on the part of the individual, facilitation of physical activity becomes the responsibility of health care professionals and depends on their ability to be encouraging, supportive, to give clear-cut information and an acceptable physical activity regimen. Every effort should be made to involve the patients and hand over responsibility.

Fear, as in ‘fear of injury’ is probably related to fear of movement,^28^ or kinesiophobia,^29^ which in turn is associated with avoidance of physical activity so as to minimize the risk of injury or re-injury. Avoidance of accident or trauma to the leg through safety thinking, precautions, compression treatment and clothing is an important issue in preventing leg ulcer. However, fear becomes dysfunctional as soon as it goes beyond safety thinking and precautions. This was seen in those individuals who felt that physical activity was a risk-taking action, as in ‘fear of injury’. Patients postponed engagements in daily activities and physical activity until ulcers had healed, with a negative effect on wound healing, functional recovery and general health. On the other hand, the individuals in both the categories ‘self-management’ and ‘a wish to stay normal’ seemed highly motivated to exercise, albeit for very different reasons.

Unluckily, exercise behaviour in individuals in the category ‘a wish to stay normal’ may increase the risk of leg trauma because of low adherence to compression therapy and a low level of safety thinking. Some of these individuals even perceive compression therapy as harmful. This is worrying, since Jull *et al.*^30^ highlighted two factors associated with compression therapy for patients whose leg ulcers healed. One was a belief that compression treatment was worth while and the other that it was beneficial in preventing ulcers.

The ‘self-management’ individuals demonstrated strong belief in their ability to be physically active despite barriers – thus relating to self-efficacy.^31^ These individuals might be able to stay physically active in a responsible way as long as they get clear-cut information and advice enabling them to adapt physical activity to the course of the disease.

### Methodological considerations

We used several strategies to ensure trustworthiness (validity) in the study. The fact that all three researchers were physiotherapists with different expertise and experience ensured that data were approached from different perspectives. The interviewer was familiar with the patient group; the second researcher had extensive experience in qualitative research and the third was a university lecturer with long-standing research experience. The research process was peer-reviewed by a third-party expert researcher in physiotherapy.

The participants had no relationship with the interviewer and were not in any position of dependence. One important question is whether the full variation of perceiving and understanding physical activity was captured in the categories described. We felt that no new information was revealed during the last interviews and therefore judged that the 22 interviews were sufficient to capture satisfactory variations in understanding of physical activity in this patient group. This is in line with recommendations in phenomenography.^32^

The weakness of qualitative research in general is that the results cannot be generalized into other contexts and people. Still we believe that our findings point out areas of importance for understanding patients’ perceptions of physical activity in venous leg ulcer. One might argue that a strategic, purposeful sampling would have been preferred in the present study to secure a wide variation in perceptions of the phenomenon studied. As many patients declined participation, the results may be preferably valid for patients holding a special interest in physical activity in relation to venous leg ulcer. However, the patients who accepted the invitation to participate represent a wide range with regard to important factors such as gender, age, BMI, previous history of leg ulcer and length of leg ulcer disease, which secures variation of perceptions and richness of detailed information.

A weakness of the present study is its relatively limited setting. For example, patients treating their wounds at home were not represented. All respondents were recruited from the patient register of an outpatient dermatology clinic. In Sweden, most patients with uncomplicated venous leg ulcer are treated by general practitioners and/or nurses from community health care and home health care organizations. Patients with poorly healing wounds, recurrent wounds, or wounds related to more complicated aetiology are referred to outpatient dermatology clinics.^33^ Thus, our approach revealed detailed descriptions from patients with experiences of often severe venous leg ulcer concerns combined with different interest in and experiences of physical activity, which seem to be valuable information as a base for improvement of care.

In summary, the perceived utility of physical activity in the context of venous leg ulcer seems to be based on (1) whether the individual understand the chronic nature of venous leg ulcer, and (2) whether the individual confront or avoid barriers experienced in relation to physical activity. On this basis the following clinical implications can be claimed:

- Patients must be given clear-cut information about the chronicity of venous leg ulcer and effect of physical activity.
- Physical activity should be prescribed as an essential treatment strategy, adapted and adjusted to the patients’ resources.
- Dysfunctional fear-avoidance beliefs should be identified and the patient guided through the avoided movements and activities.
- Knowledge of the wide range of perceptions of physical activity in this patient group might enhance care givers to design physical activity regimens that reflect the individuals’ perceptions.

## Clinical message

The perceived utility of physical activity in the context of venous leg ulcer seems to be based on: (1) the perception of venous leg ulcer as a chronic or acute condition and (2) engagement or avoidance behaviour toward physical activity.
